# Persian language health websites on Ebola disease: less credible than you think?

**DOI:** 10.1186/s42506-019-0027-4

**Published:** 2020-01-29

**Authors:** Masoumeh Ansari, Ronak Hamzehei, Saeideh Valizadeh-Haghi

**Affiliations:** 1 Clinical Research Development Unit, Kowsar Hospital, Kurdistan University of Medical Sciences, Sanandaj, Iran; 2 Clinical Research Development Unit of Shahid Beheshti Hospital, Hamadan University of Medical Sciences, Hamadan, Iran; 3 Department of Medical Library and Information Sciences, School of Allied Medical Sciences, Shahid Beheshti University of Medical Sciences, Tehran, Iran

**Keywords:** Ebola, Health portals, Patient education, Health websites, Website evaluation, Health information

## Abstract

**Background:**

Ebola virus disease is an emergency situation in the area of international public health for which currently, there is no standard treatment. Thus, there is an essential need for awareness of individuals about the Ebola disease and consequently its prevention. Internet and health websites are considered a source of health information about certain diseases. Therefore, in this study, the credibility of Persian-language websites on Ebola is assessed.

**Materials and methods:**

The term “Ebola” was searched using the Yahoo, Google, and Bing search engines. The first 30 websites resulting from each search engine were studied. Persian language was a prerequisite for inclusion. Duplicate and inaccessible websites were excluded and 62 websites were retained for evaluation. These websites were manually assessed by the researchers. The research tool was HONcode official toolbar as well as the checklist set by the researchers based on the HONcode of Conduct tool. The data were analyzed using SPSS 18.0 software.

**Results:**

None of the retrieved websites was officially approved by the HONcode of Conduct. Moreover, the manual evaluation showed that none of them had fully considered the eight criteria of HONcode. The results showed that most of the retrieved websites (62%) were commercial. The justifiability criterion had been considered in 89% of the studied websites, but the authority criterion had been considered by only 16% of the websites.

**Conclusion and recommendations:**

Regarding the poor reliability of Persian websites on the Ebola disease, and considering that Persian language people prefer to read the information in their native language, it is recommended that the authorized health organizations introduce reliable health websites in the Persian language. This will help them to take part in active healthcare decision-making and disease prevention. Moreover, it is necessary to educate people especially Persian language ones about the website evaluation tools, which can be used to assess the credibility of health websites before consuming the information on those websites.

## Introduction

The Ebola virus disease is an emergency situation in the area of international public health [1]. This disease emerged in West Africa in late December 2013 and led to a massive outbreak in places such as Guinea, Liberia, Sierra Leone, and Nigeria [2]. Ebola is a serious and often fatal illness in humans [3] for which currently, there is no standard treatment [2] and no vaccine has been produced to prevent it. Given that prevention is always better than cure, the need for people’s awareness of the disease, how it is developed, and its prevention methods is critical.

The Internet has become one of the main and the most popular sources of health information in recent years [4, 5]. Most people throughout the world refer to the Internet and health websites to obtain information about certain diseases, treatments, diets, fitness, and other medical issues [5, 6]. Health information is one of the three highly functional and favorite subjects of users on the Internet [6]. Accordingly, 4.5% of all Internet searches worldwide are related to health topics [7]. This media provides easy and increasing access to health information for the general public. Users of these information often refer to the information available on the Internet before meeting with health professionals or after receiving medical advice [7, 8]. But how much can we trust the information contained on such websites? This issue is more important in medicine, where the quality of information affects human life.

The quality of retrieved information is a matter of concern due to the lack of control over the content of information published on the Internet. Various researches conducted on the quality of health websites in various topics showed that, in most cases, the quality of health-related websites is poor and needs further attention [9–15]. No authority is directly responsible for managing Internet resources. Therefore, dissemination of information on the web is easy and inexpensive, or even free, and any person with any level of expertise can publish his/her health information resources on the web with any degree of credibility. So, a wide variety of information of varying quality and by authors of varying degrees of reliability is made available. As noted by Silberg and colleagues, “It is a medium in which everyone with a computer can serve simultaneously as author, editor, and publisher” [16]. Therefore, recognizing the credibility of health websites is of the utmost importance.

Some measures should be taken to review the websites and ensure the quality of retrieved health information via the Internet. In this regard, various tools have been created by different organizations to evaluate the quality of health websites in order to help users and website founders. Among these tools are the HONcode of Conduct, Silberg criteria, Discern, Hi-Ethics, and AMA guidelines. HONcode of Conduct is one of the tools that was created in 1995 and have been frequently used to evaluate the quality of health information websites [10–15, 17–22]. It guides lay users as well as health professionals to validate medical resources on the Internet [23].

Persian is the second language in the Middle East, and about 110 million people speak the language in the world. The language is an Indo-European language, spoken and written primarily in Iran, Afghanistan, and a part of Tajikistan. A significant proportion of Persian speakers live in other countries, including Iraq, the UAE, and Bahrain. In Persian-speaking countries like other countries, the internet is one of the tools which are used to obtain health information. The importance of the phenomenon of the Persian web as an independent and effective media in the communication of Persian speakers is undeniable. Limited English proficiency affects effective health communication [24]. Furthermore, language provides the experiential context for understanding health information [25] because patients with limited English proficiency have restricted ability to read or understand English materials [26]. Certainly, reading the health educational materials in vernacular language is more understandable.

In this regard, the necessity to assess the websites in the Persian language is of great importance due to their direct impact on the health of Persian-language society members. Therefore, considering the Ebola virus as one of the most important health challenges in the world, in this study, the Persian-language websites which have published information in the field of Ebola are evaluated using the HONcode tool.

## Materials and methods

**Fig 1.**
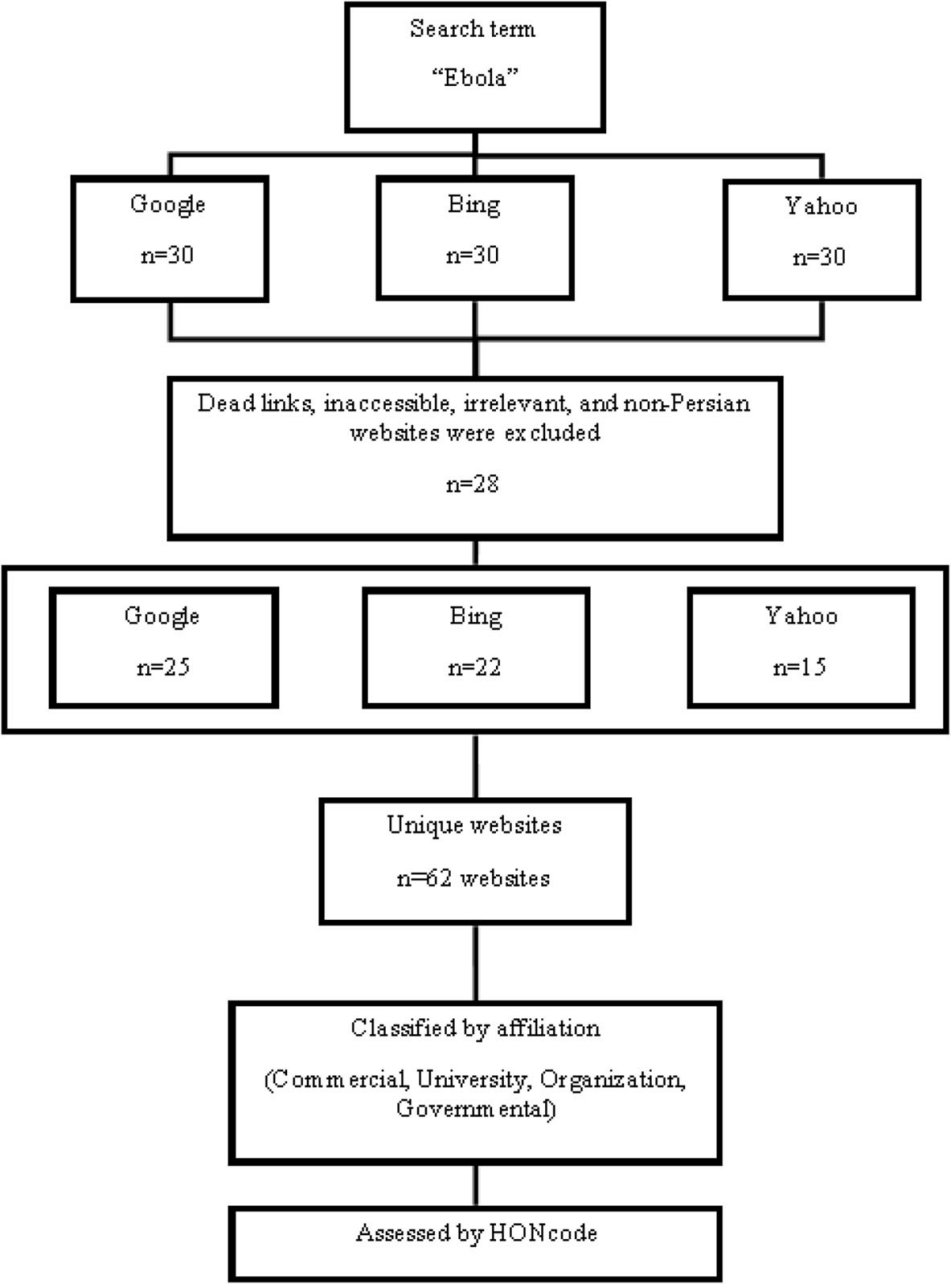
Data collection process

Search engines are the first and the most important tools and the resource of information for users of the Internet [27]. They play an important role in obtaining health information by non-specialists [28]. According to the Alexa website statistics, Google, Yahoo, and Bing are three of the most widely used search engines in the world [29, 30]. Thus, for this study, the term “Ebola” (In Persian language and with the Persian alphabet) was searched using these three most used search engines [31]. The Chrome browser was used in this search. Given that 90% of search engine users review and study one or some of the obtained results in the first three pages of search results [32, 33], the first 30 websites resulting from the search in the three selected search engines, with a total of 90 web pages, were studied. After excluding 28 websites including duplicate, inaccessible, and non-Persian websites, 62 websites were retained for evaluation. The flow chart that describes how the data were collected and processed is shown in Fig. 1.

In the first step, the retrieved websites were divided into four categories: university, governmental, commercial, and organizational. Then, they were manually assessed by MA and RH. The validity of the data was assessed by SV. The search and data collection was conducted using a direct observation method on February 9, 2017. The research tool was the checklist set by the researchers based on the HONcode of Conduct tool. This tool consists of eight criteria and these are authority, complementarity, privacy, attribution, justifiability, transparency, financial disclosure, and advertising policy (Table 1). The data were analyzed using SPSS 18.0 software.

**Table 1:** HONcode principles*

HONcode principles	Description
1. Authoritative	Indicate the qualifications of the authors
2. Complementarity	Information should support, not replace, the doctor-patient relationship
3. Privacy	Respect the privacy and confidentiality of site users
4. Attribution	Cite the source[s] and dates of published medical information
5. Justifiability	Site must back up claims relating to benefits and performance
6. Transparency	Accessible presentation, accurate email contact
7. Financial disclosure	Identify funding sources
8. Advertising policy	Clearly distinguish advertising from editorial content

*The table information is adopted from the HON website [23]

## Results

The data collection and frequency of the unique websites retrieved by Bing, Yahoo, and Google are shown in Fig. 1. Of the 90 webpages retrieved by 3 used search engines, 62 websites were retained for evaluation. In terms of uniqueness, the Google search engine had the highest frequency among the search engines.

**Fig. 2.**
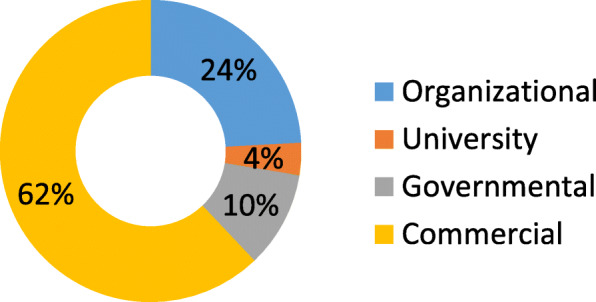
Distribution of websites by organization

As shown in Fig. 2, most of the retrieved websites (62%) were commercial and only 4% of the retrieved websites were provided by universities.

Overall, none of the retrieved websites was officially approved by the HONcode of Conduct. Moreover, the manual evaluation showed that none of them had fully considered all eight criteria of HONcode (Table 2). In general, the justifiability criterion had been considered in 89% of the studied websites, but only 16% of the websites have identified the name and expertise of the author. The authority criterion was highly considered (20%) by the websites retrieved by Google search engine. The websites retrieved by the Bing search engine had the highest frequency in considering the attribution, justifiability, complementarity, and financial disclosure criteria. The advertising policy, transparency, and privacy criteria had been less considered by the websites retrieved by Yahoo (Table 2). In general, and considering all the criteria, it was found that 51% of the criteria were met by the assessed websites.

**Table 2:** Total number and Percentage of websites which are in compliance with each criterion of HONcode

Criteria	Search Engines			Total
	Yahoo *N* = 15	Google *N* = 25	Bing *N* = 22	
Authority	2 [13%]	5 [20%]	3 [14%]	10 [16%]
Complementarity	12 [80%]	19 [76%]	18 [82%]	49 [79%]
Privacy	10 [66%]	16 [64%]	13 [59%]	39 [63%]
Attribution	6 [40%]	10 [40%]	14 [64%]	30 [48%]
Justifiability	12 [80%]	21 [84%]	22 [100%]	55 [89%]
Transparency	4 [27%]	5 [20%]	4 [18%]	13 [21%]
Financial disclosure	6 [40%]	7 [28%]	9 [41%]	22 [35%]
Advertising policy	9 [60%]	13[52%]	13 [59%]	35 [56%]
Total	61/120 [51%]	96/200 [48%]	96/176 [54%]	253/496 [51%]

Since every website has to comply with eight criteria in terms of HON code criteria, so in general, for reviewed websites of Google (25 × 8 = 200), Yahoo (15 × 8 = 120), and Bing (22 × 8 = 176) criteria should ideally be met. While the results showed that of all the criteria, websites retrieved from the Google search engine have met 96 (48%), Yahoo 61 (51%), and Bing 96 (54%) of the criteria.

## Discussion

The manual evaluation of Persian language websites showed that the Ebola-related websites were of poor credibility because none of those websites met all the eight HONcode criteria (Table 2). The results of this study were consistent with the research results of Ebola English-language websites [34]. Furthermore, the results of the present study were consistent with the results of similar studies conducted on the evaluation of non-Persian language websites in other health topics. Their findings showed unreliability and poor quality of health websites [34–37].

None of the Persian language websites evaluated at the present study was officially approved by the HONcode foundation, while the study on English language websites on Ebola showed that seven websites were approved officially [34]. It is not out of the mind that this lack of credibility exists in Ebola-related websites in other languages, and that the websites do not have good credibility, although there is a need for detailed studies in this regard. Therefore, users must act with full awareness when using the Persian-language websites as a source of health information regarding Ebola, and they must use the provided information alongside expert medical advice from relevant experts [11].

In the current study, overall, the justifiability criterion obtained the highest score (Table 2). These findings show that the majority of the websites related to Ebola diseases try to present the correct information and the actual performance of a particular treatment, drug, or medical device, and their related consequences. However, still, there is lack of honesty in expressing material or commercial use in 11% of websites.

The authority criterion obtained the lowest score (Table 2). Only 16% of the websites had specified the name and expertise of authors. However, the compliance with this criterion reflects the credibility and reliability of the information resource, because this principle proves that health information provided on the intended website that has only been given by qualified professional resources and written by experts is more reliable [38].

The complementarity criterion has been considered in most of the surveyed websites (79%). The compliance with this criterion shows that the surveyed websites try to guide the people to visit a physician instead of making health decisions just based on online information [23]. Nevertheless, still, 21% of surveyed websites have not paid attention to this criterion that may lead to the misuse of information.

The website must describe its privacy policy regarding how it treats confidential or private information such as email addresses and the content of emails received from or sent to its visitors. However, just 63% of the surveyed websites have identified privacy policy. While privacy policy is one of the seven key usability design issues that are particularly important for creating effective websites [39].

Transparency principle is considered in just 21% of the surveyed websites. This principle indicates that in case of requiring additional information, people must be able to connect with content editors and to communicate with webmasters. Regarding that 79% of the Ebola-related websites have not considered this principle, it is necessary to website owners to modify their websites to help individuals to get additional information.

Attribution policy has been considered in just 48% of the surveyed websites. It is necessary to specify the publication date as well as the latest updating of the content. This principle, in fact, helps to ensure the credibility of the written medical content. Medical content must continually be updated, in the event of non-compliance with this principle, the date is not revealed. Regarding that 52% of the Ebola-related websites had not identified the source of information or the last update date, people should be aware of the unreliable or outdated information. Therefore, it is recommended that the health websites and consequently health information retrieved by general search engines be used with more caution.

The advertising should be clearly distinguished from editorial contents, but advertisement policy was less considered in the surveyed websites (56%) which was mostly retrieved by Google search engine. Failure to comply with this principle indicates that the individuals may not be able to distinguish the advertisement information from the main content. Thus, their trust in marketing information may mislead them to unreliable information which threatens their health.

The health websites including all types of websites (organizational, commercial, governmental, etc.) must include the declaration of the funding resources as well as the declaration of all conflicts of interest. While in the present study, just 35% of the surveyed websites have declared financial disclosure. Failure to comply with this principle indicates that the website may recommend the various medical interventions that have a hidden marketing aspect. Thus, encouraging the individuals to use medication or a therapeutic method, and the individual’s trust in such information, may have irreparable consequences for them.

According to the results obtained from each search engine, Google, Yahoo, and Bing, it was found that none of the evaluated websites had considered all eight criteria. In terms of considering the authority criteria, amongst the three selected search engines, the websites returned by Google were in better condition (Table 2). Regarding the complementarity, attribution, justifiability, and financial disclosure criteria, it seems that the webpages retrieved by Bing are in better condition than Yahoo and Google. Regarding the advertising policy, transparency, and privacy principles, the webpages retrieved by Yahoo are in better condition. Meanwhile, the status of websites retrieved from the Bing search engine was generally better than other search engines and Google was worse than the others, although the difference was negligible (Table 2).

Most of the retrieved websites in this study (Fig. 2) were commercial [62%], similar to the results reported in other studies conducted to evaluate the health websites [17], though commercial websites have lower quality compared to other websites [22, 40–42]. The government and university websites generally are aimed to educate people [43]. In this study, 10% of retrieved websites were of governmental type. Furthermore, just 4% of the retrieved websites were of the university type. The university websites, which a person would tend to trust more, showed no significant advantages in credibility compared with other types of websites. While the university websites are expected to highly consider the HONcode of conduct principles, but the present study findings revealed that they are not so, as the findings of studies on other health topics [34, 44]. Thus, looking for health information on Ebola, the individuals may encounter less credible websites which may include information that is more commercial than educational. Consequently, they may receive information that is harmful to their health. Moreover, in this study, 24% of the retrieved websites were organizational type in which patients can access to some records on health issues such as future appointments, history of past visits, and the patient laboratory results. This type of website also did not show any significant advantages in reliability compared with other types of websites. Thus, individuals searching for Ebola-related information must use all types of health websites with caution.

In the current study, the 62 evaluated Persian websites are samples of the websites that Persian language users will face when searching for information about Ebola. Failure to comply with all HONcode criteria in these websites shows that while searching on the Internet, Persian language users will encounter none credible websites and consequently low-quality information that can affect their decision about the prevention and treatment of Ebola. This highlights the importance of understanding the quality of online health information by physicians and how to guide patients to reliable sources and high-credible websites [45].

This study was faced with some limitations. The “Ebola” keyword was searched in April 2016. Therefore, this study cannot completely and comprehensively represent other searches at different times. On the other hand, due to the dynamic characteristics of the web, the results of the search will vary at different times and in different places. New websites are constantly created, while some websites are disbanded. On the other hand, the present study was conducted only on Persian-language websites. The results of this study may be different from the results of studies conducted in other languages, although the previous research by the researcher on English-language websites was consistent with the results of this study.

## Conclusion and recommendations

Regarding the poor reliability of Persian websites on the Ebola disease, and considering that Persian language people prefer to read the information in their native language, it is recommended that the authorized health organizations introduce reliable health-related websites in the Persian language. This can prevent the use of unauthorized websites by individuals and the use of inaccurate information to some extent. Moreover, this will help them to take part in active healthcare decision-making and disease prevention.

Moreover, it is necessary to educate people especially Persian language ones about the evaluation tools, which can be used to assess the credibility of health websites before consuming the information on those websites.

## Data Availability

Data available on request from the authors.
